# Split-GFP lamin as a tool for studying *C. elegans* LMN-1 dynamics *in vivo*

**DOI:** 10.17912/micropub.biology.001022

**Published:** 2023-12-13

**Authors:** Ellen F. Gregory, James Matthew Ragle, Jordan D. Ward, Daniel A. Starr

**Affiliations:** 1 Department of Molecular and Cellular Biology, University of California, Davis, Davis, California, United States; 2 Department of Molecular, Cell, and Developmental Biology, University of California, Santa Cruz, Santa Cruz, California, United States

## Abstract

We engineered a fluorescent fusion protein of *C. elegans* lamin, by fusing the eleventh beta strand of GFP to the N-terminus of LMN-1 at the endogenous *lmn-1* locus. When co-expressed with GFP1-10, GFP11::LMN-1 was observed at the nuclear periphery of a wide variety of somatic cells. Homozygous *gfp11::lmn-1* animals had normal numbers of viable embryos. However, the *gfp11::lmn-1* animals had a mild swimming defect. While not completely functional, the GFP11::LMN-1 strain is more healthy than other published fluorescent LMN-1 lines, making it a valuable reagent for studying lamins.

**Figure 1. GFP11:: LMN-1 localization and function:**
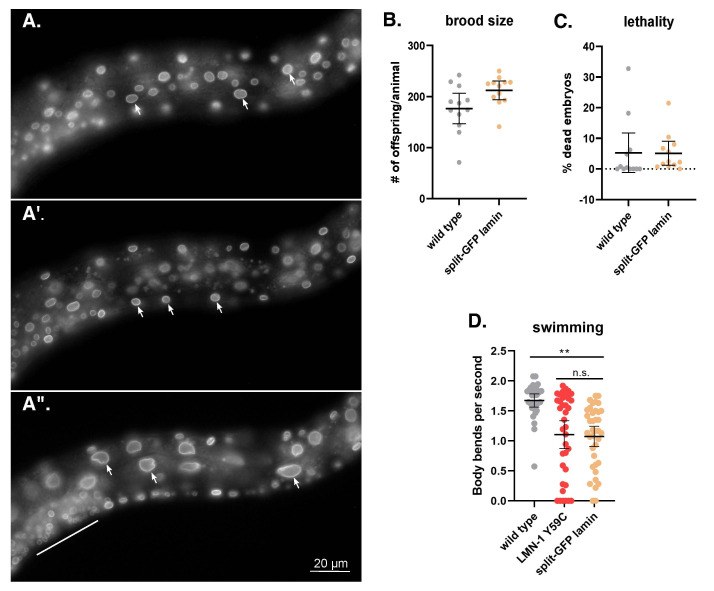
(A) GFP fluorescence is shown in three different focal planes of an L4 larvae. A lateral view is shown with ventral down and anterior to the left. The scale bar is 20 mm. GFP localizes to the periphery of most somatic cells, including cells from the ventral hypodermis (arrows in A), body wall muscles (arrows in A’), and intestine (arrows in A”). GFP can also be seen in many other cells, including the developing vulva (above the line in A”). (B) The brood size and, (C) the percent of embryonic lethality in wild type and GFP11::LMN-1 (split-GFP lamin) animals. Each data point represents the brood from a single adult. The means are shown with error bars of 95% CI. Significance was calculated using student’s t-test. (D) The swimming rate, measured in body bends per second, of wild type, *lmn-1 (Y59C)* and split-GFP lamin in L4 larvae. Means and error bars of 95% CI are shown. n=40. Significance was calculated using a one-way ANOVA and corrected for multiple comparisons by Tukey HSD. **p≤0.01; n.s.= not significant.

## Description

Lamins are intermediate filament proteins that form a structural meshwork just inside the inner nuclear membrane. They provide structure to the nuclear envelope and organize chromatin (Turgay et al. 2017; Leeuw et al. 2018; Tenga and Medalia 2020) . Defects in lamins cause a wide variety of diseases called laminopathies including muscular dystrophies with cardiac defects, lipodystrophies, and premature aging syndromes (Shin and Worman 2021; Storey and Fuller 2022) . Given these critical roles in normal cell and developmental biology as well as in disease, lamins are versatile, multifunctional, and challenging proteins to study. *C. elegans* is a popular model to study the cellular and developmental roles of lamin (Lyakhovetsky and Gruenbaum 2014; Cohen-Fix and Askjaer 2017; Charar et al. 2021) . *C. elegans* has a single lamin, LMN-1 , similar in form and function to both B- and A-type lamins but often categorized as a B-type lamin because it is broadly expressed throughout development and is constitutively farnesylated at its C terminus (Liu et al. 2000; Bar et al. 2009; Dittmer and Misteli 2011) . The well-established genetic tools available and the transparency of *C. elegans* throughout their development offers an opportunity to examine cellular processes in live animals. In addition, *in vivo* imaging using fluorescent tags can be used to study protein function, making *C. elegans* an excellent system for studying nuclear dynamics (Fridolfsson and Starr 2010; Sarov et al. 2012; Heppert et al. 2016; Bone et al. 2016; Cohen-Fix and Askjaer 2017; Breimann et al. 2019) .

Despite the advantages described above, live imaging of lamins in *C. elegans* has proved challenging. One hurdle is that lamins multimerize to form higher-order filaments (Turgay et al. 2017) , making it difficult to tag endogenous versions of lamins with fluorescent proteins without disrupting their function. Early GFP:: LMN-1 models expressed GFP-tagged wild-type and point mutants from multi-copy extrachromosomal arrays (Wiesel et al. 2008; Mattout et al. 2011; Zuela et al. 2016) . While they localized to the nuclear periphery as expected, they were expressed at unknown levels along with endogenous LMN-1 . More recently, CRISPR/Cas9 gene editing has been used to insert coding sequences for GFP directly into the endogenous *lmn-1* locus. When the C-terminus of LMN-1 is tagged with GFP, it leads to 100% nonviable progeny (Bone et al. 2016) . Animals expressing an N-terminally tagged GFP:: LMN-1 from the endogenous *lmn-1* locus exhibit less severe brood size and lethality defects, but are still significantly less healthy than wild-type animals, laying about half as many eggs, of which only 65% are viable (Bone et al. 2016) . An alternative to tagging LMN-1 at its ends is to tag it in the middle. Insertion of an auxin-inducible degron into the middle of LMN-1 has no apparent effect on the viability of animals (Liu et al. 2023) .

To circumvent the lethality of attaching a full-length GFP tag to lamin, we developed a split-GFP lamin transgenic line, a technique recently developed in *C. elegan* s (He et al. 2019; Goudeau et al. 2021) . We used CRISPR/Cas9 to insert a sequence encoding the 11 ^th^ beta strand of GFP (GFP11) into the endogenous *lmn-1* locus (GFP11:: LMN-1 ; see Materials and Methods). The remaining ten beta strands (GFP1-10) were somatically expressed from a single-copy GFP1-10 transgene (Costa et al. 2023) . A complete GFP that fluoresces normally forms following the interaction of all eleven beta strands within the nucleus (Kamiyama et al. 2016) , allowing for observation of endogenous lamin structures.

The functionality of GFP11:: LMN-1 was tested by assaying protein localization, progeny viability, adult fecundity, and animal movement. GFP11:: LMN-1 localized as expected to the nuclear periphery in cells that also expressed GFP1-10. We observed strong GFP localization to the periphery of nuclei in somatic tissues broadly expressed across all three germ layers, including the hypodermis ( Figure 1A ), body wall muscles ( Figure 1A ’) and the intestine ( Figure 1A ”). GFP11::LMN-1 was not detected in the germline, probably because the GFP1-10 transgene we used is not expressed in the germline. We encourage someone in the field to cross the GFP11::LMN-1 line into a strain with GFP1-10 expressed in the germline to follow LMN-1 in the early embryo. GFP11:: LMN-1 animals had no viability or fecundity defects ( Figure 1 B -C). However, we did observe a significant swimming defect ( Figure 1D ). This decrease in swimming activity was equivalent to defects previously described in strains with LMN-1 mutants associated with disease , including the Y59C variant associated with skeletal and cardiac muscle disease (Wiesel et al. 2008; Mattout et al. 2011; Zuela et al. 2016; Gregory et al. 2023) . Nevertheless, our GFP11:: LMN-1 strain is the least disruptive GFP-lamin construct developed to date and should therefore be a useful tool to the field.

## Methods

The assays for brood size, embryo viability, and swimming activity were performed exactly as previously described (Gregory et al. 2023) . Images were taken with a wide-field epifluorescence Leica DM6000 microscope with a 63 × Plan Apo 1.40 NA objective, a Leica DC350 FX camera, and Leica LAS AF software version 3.7.3.

Strains JDW182 ( *bmd15 [* eef-1A.1p::GFP1-10::unc-54 3'UTR; myo-2 p::mCherry::3xHA::tbb-2 3' UTR *] I, lmn-1 ( wrd39 [1xGFP11:: lmn-1 ])* I) and DQM104 ( *bmd15 [* eef-1A.1p::GFP1-10::unc-54 3'UTR; myo-2 p::mCherry::3xHA::tbb-2 3' UTR *] I* ) (Costa et al. 2023) will be available at the Caenorhabditis Genetics Center, which is funded by NIH Office of Research Infrastructure Programs (P40 OD010440). Strain UD833 ( *lmn-1 (yc96[Y59C]) I/hT2 (I;III); him-8(e1489) IV; ycIs10 V* ) (Gregory et al. 2023) is available on request.

CRISPR/Cas9 mutagenesis was performed as previously described (Paix et al. 2014; Dokshin et al. 2018; Morrison et al. 2021) . JDW182 animals were gene edited by the insertion of a sequence coding for GFP11 after the initiation codon of the endogenous *lmn-1* gene (Kamiyama et al. 2016; He et al. 2019) . The edit was performed in a strain containing a strongly expressed *eef-1A.1p::GFP1-10* transgene (Costa et al. 2023) . DQM104 animals were injected with a Cas9 ribonucleoprotein complex consisting of 250 ng/μl of house-made Cas9 protein (Zuris et al. 2015) , 20 ng/μl crRNA (5’-AGAAAAATGTCATCTCGTAA-3’; IDT), and 44 ng/μl tracrRNA (IDT). The mixture was first incubated at 37°C for 15 minutes, then mixed with the coinjection marker pRF4 (50 ng/μl) and repair oligos ssDNA #4288 (110 ng/μl). We injected into animals that were picked as L4s and aged one day at 20ºC. Four days post-injection we screened wells containing a large percentage of rolling animals for GFP positive animals. We singled 12 GFP+ WT-moving L4s from a single Rol GFP+ well, incubated them at 25ºC and scored for wells with 100% GFP progeny that had lost the Rol co-injection marker.

We confirmed the knock-in through PCR genotyping and Sanger sequencing using the following oligos in *lmn-1* : ggaatactgccatctgccga and tggcaagacgactgttgagt. For the repair template, we used Oligo #4288: tataattaactcttcagaaagcagcgagaaaa atg **CGTGACCACATGGTCCTCCACGAGTACGTCAACGCCGCCGGAATCAC** CGGAGGATCCGGAGGAtcatctcgtaaaggtactcgtagttctcgtattgttacgctagagcgct. Lowercase sequences have homology with the 5’UTR or first exon of *lmn-1 .* The underlined atg is the endogenous start codon of *lmn-1* . The upper-case sequences are the inserted sequences. The bold sequences code for GFP11 and the non-bold upper-case sequences code for a linker peptide of GGSGG residues.

## Reagents

**Table:** 

strain	genotype	source
JDW182	*bmd15 [* eef-1A.1p::GFP1-10::unc-54 3'UTR; myo-2 p::mCherry::3xHA::tbb-2 3' UTR *] I, lmn-1 ( wrd39 [1xGFP11:: lmn-1 ])* I	this study
DQM104	*bmd15 [* eef-1A.1p::GFP1-10::unc-54 3'UTR; myo-2 p::mCherry::3xHA::tbb-2 3' UTR *] I*	Costa et al. 2023
UD833	*lmn-1 (yc96[Y59C]) I/hT2 (I;III); him-8(e1489) IV; ycIs10 V *	Gregory et al. 2023
